# The synergistic hepatoprotective potential of *Beta vulgaris* juice and 2,3- dimercaptosuccinic acid in lead-intoxicated rats via improving the hepatic oxidative and inflammatory stress

**DOI:** 10.1186/s12906-020-03056-6

**Published:** 2020-09-01

**Authors:** Nadia Z. Shaban, Samah A. Abdelrahman, Mohamed A. L. El-Kersh, Fayed A. K. Mogahed, Iman M. Talaat, Noha H. Habashy

**Affiliations:** 1 Biochemistry Department, Faculty of Science, Alexandria University, Alexandria, 21511 Egypt; 2 Department of Nucleic Acid Research, Genetic Engineering and Biotechnology Research Institute, City of Scientific Research and Technological Applications (SRTA-City), New Borg EL-Arab, Alexandria, 21934 Egypt; 3 Department of Pathology, Faculty of Medicine, University of Alexandria, Alexandria, Egypt

**Keywords:** *Beta vulgaris* juice, 2,3- dimercaptosuccinic acid, Lead hepatotoxicity, Oxidative stress, Inflammation, Synergism

## Abstract

**Background:**

Lead (Pb) is observed in all areas of the environment, mainly derived from human operations such as mining, processing, and burning fossil fuels. Pb toxicity is one of the most prevalent causes of human hepatotoxicity. The available chelator drugs used now have many adverse effects and therefore the world is looking for natural and secure alternatives.

**Methods:**

Here, we evaluated the hepatoprotective role of the oral administration (1 g/kg b.w.) of the lyophilized *Beta vulgaris* juice (BVJ) against Pb-induced rat hepatotoxicity. We also examined the possible synergistic hepatoprotective impact of the combination between BVJ and 2,3- dimercaptosuccinic acid (DMSA, the currently approved drug for Pb-toxicity). The evaluation depends on the ability of BVJ, DMSA, or their combination (BVJ-DMSA) to reduce serum and hepatic Pb level and to avoid oxidative stress and inflammation caused by Pb. The level of lipid peroxidation, reduced glutathione (GSH), total antioxidant capacity, and the activity of the antioxidant enzymes were quantified. In addition, the level of interleukin (IL)-6, nitric oxide (NO), DNA fragmentation, and liver histology were studied.

**Results:**

The results showed that BVJ contained considerable amounts of betalains, vitamin C, and various types of phenolic compounds. Therefore, BVJ displayed a significant (*p* < 0.05) preventive influence on the elevation of Pb levels in blood and liver as well as the hepatic DNA fragmentation. In addition, it significantly (*p* < 0.05) improved most of the studied antioxidant and inflammatory markers in the Pb-intoxicated rats. However, the combined extract (BVJ-DMSA) revealed synergistic (combination index < 1) activities in most of the tested parameters. The histopathological results verified the biochemical findings of this research.

**Conclusion:**

BVJ has a potent efficiency in the protection from Pb-induced hepatotoxicity through the reduction of its accumulation in blood and liver and the prevention of the oxidative stress and inflammation induced by Pb. Additionally, the treatment of hepatotoxicity with BVJ and DMSA in combination showed a synergistic effect and reduced the adverse effects induced by DMSA. Thus, BVJ can be a promising hepatoprotective extract against lead toxicity and its combination with DMSA potentiates this effect.

## Background

The liver is the largest internal organ and gland of the human body. It has a weight of 1.4–1.6 kg in the adult, which represents about 2% of the whole-body weight. It has several functions in the body such as metabolism of lipids, carbohydrates, and proteins, production of coagulation factor and albumin, as well as storage of vitamins and glycogen. In addition, it detoxifies xenobiotics and drugs, so it can expose to several diseases including toxicity [1]. Hepatotoxicity can also be induced by natural chemical agents (such as aflatoxin), industrial agents, and heavy metals (such as lead, Pb) [2]. The Pb is a toxic heavy metal with a wide abundance in the earthand it can be absorbed via the respiratory system, gastrointestinal tracts, and rarely through the skin [3]. After absorption, Pb is distributed in the blood, bone, and soft tissues, while the liver is the main storehouse (33%). It can interact with the cellular macromolecules causing proteins dysfunction, lipid peroxidation, DNA damage, and oxidative stress (OS) [2]. OS is an imbalance between free radicals [reactive oxygen (ROS) and nitrogen (RNS) species] and antioxidants [4]. OS induces hepatocyte inflammation, proliferation, necrosis, loss of hepatic reticular fiber, fatty changes, and mild fibrosis [5].

Chelation therapy is a conventional way to treat heavy metal poisoning [6] by creating an insoluble, less toxic metal-complex [7] that can easily be excreted from the body [6]. 2,3-dimercaptosuccinic acid (DMSA, succimer) is the ideal chelating agent for Pb in children and sensitive adults [8]. However, it has several adverse effects, including increased levels of alanine aminotransferase (ALT) and aspartate aminotransferase (AST) [6] and is unable to remove Pb from hard tissues such as bones. Therefore, the use of DMSA in chronic cases of Pb injury is limited [9].

Nowadays, huge kinds of vegetables, herbs, and medicinal plants are suggested for the treatment of hepatotoxicity [10]. For the first time, the current study evaluated the protective role of Beetroot (*Beta vulgaris*, BV) juice (BVJ) against Pb-induced OS and injury to the rat liver. In addition, we studied the possible hepatoprotective synergism of the combination between BVJ and DMSA. The BV is the taproot part of the beet plant belonging to the Chenopodiaceae family and considered to be vegetable and fruit. It is distributed all over the world and has been cultured for hundreds of years in all temperate climates. The BV is an important source of nitrogenous pigments called betalains (yellow betaxanthins and red betacyanins). In addition, it contains many functional constituents such as polyphenols, vitamins, minerals, and others. Therefore, BV is considered as a medicinal plant with hepatoprotective and antioxidant effects and can be used in the treatment of several diseases [11]. In the present study, the functional constituents and antioxidant properties of BVJ were examined to explain its expected hepatoprotective action against Pb intoxication in rats.

## Methods

### Chemicals

Folin–ciocalteau reagent, 4-hydroxycinnamic acid (4-HCA), rutin (RU), catechin, butylated hydroxytoluene (BHT), 2,2- azino-bis(3-ethylbenzthiazoline-6-sulfonic acid (ABTS),monoisoamylDMSA, thiobarbituric acid (TBA), reduced glutathione (GSH), and 5,5`-{dithiobis-2-nitrobenzoic acid} (DTNB) were obtained from Sigma-Aldrich (St. Louis, MO, USA). Lead acetate was purchased from ISO-CHEM, France. Alkaline phosphatase (ALP), Albumin, ALT and AST kits were obtained from Giesse Diagnostics, Italy. Urea and creatinine kits were supplied by Diamond Diagnostics, Egypt. Easy-spin™ [DNA free] total RNA extraction kit was obtained from iNtRON Biotechnology, South Korea. SensifastsyBr lo-Rox one-step kit and MyTaq Red Mix kit were purchased from Bioline (USA). Primers and 50 bP DNA ladder kit were obtained from Vivantis (USA) and Genedirx (USA), respectively. Rat IL-6 ELISA kit was purchased from RayBiotech (Norcross, USA). Other chemicals were obtained with a high grade.

### BVJ preparation

The BV plant (NCBI:txid3555) was purchased from specific herbal stores in Alexandria, Egypt. After authentication and identification of the plant by the experts in these stores, it was pressed using a dry household juice extractor to get the juice, which was lyophilized (Telstar, Terrassa, Spain). The powdered form (yield 13 g/100 mL juice) has been stored at − 20 °C until used.

### BVJ constituents

Some constituents in BVJ, including total phenolics, flavonoids, tannins, betalains, anthocyanins as well as vitamin C, were quantified. Total phenolic compounds in mg 4-HCA equivalents/g extract were estimated colorimetrically at 750 nm after reduction of Folin-Ciocalteau reagent by juice phenolics [12]. Total flavonoids were measured at 510 nm after mixing an aliquot of BVJ with NaNO_2_ (5%) and AlCl_3_ (10%) and the concentration in mg RU equivalents/g extract was calculated using RU standard curve [13]. While the tannin content of the extract was quantified by the modified vanillin assay using the catechin calibration curve [14]. Total betalains (betacyanins and betaxanthins) were quantified by measuring the diluted solution of BVJ at three different wave lengths, 536 nm, 485 nm, and 650 nm (impurities) [15]. The concentration of betacyanins or betaxanthins has been calculated using the following equation:

$$ \left[\mathbf{Betacyanins}\ \mathbf{or}\ \mathbf{betaxanthins}\ \left(\mathbf{mg}/\mathbf{L}\right)=\frac{\boldsymbol{A}\ \boldsymbol{x}\ \boldsymbol{M}.\boldsymbol{Wx}\ \boldsymbol{DF}\ \boldsymbol{x}\ \mathbf{1000}}{\boldsymbol{exi}}\right]. $$

Where, A = A_**536**_– A_**650**_ (for betacyanins) or A_**485**_ – A_**650**_ (for betaxanthins), M.W: molecular weight of betacyanins or betaxanthins (550 or 336 g/mol, respectively), DF: Dilution factor, ϵ: molar extinction coefficient for betacyanins or betaxanthins (60,000 or 48,000 L/molcm, respectively), i: path length (cm). Anthocyanins were quantified by a pH-differential assay that based on the reversible structural transformation of these pigments due to pH change [16]. The sample was mixed with two buffer solutions (pH 1.0 and pH 4.5) and the absorbance has been measured at 510 and 700 nm for each pH. The absorbance of the sample (A_s_) was calculated from the equation: [**As =** (**A**_**510**_ **− A**_**700**_)_**pH 1.0**_ **−** (**A**_**510**_ **− A**_**700**_)_**pH 4.5**_] Whereas the anthocyanins concentration was determined as cyanidin-3-glucoside (Cy-3-glc) equivalent using the equation:

$$ \left[\mathbf{anthocyanin}\ \mathbf{pigment}=\frac{\left({\mathbf{A}}_{\mathbf{s}}\times \mathbf{MW}\times \mathbf{DF}\times \mathbf{1000}\right)}{\left(\boldsymbol{\upvarepsilon} \times \mathbf{sample}\ \mathbf{weight}\right)}\right]. $$

Where MW is the molecular weight of Cy-3-glc and ε is its molar absorptivity, DF is the sample dilution factor.

Vitamin C concentration has been measured spectrophotometrically by 2,4 dinitrophenyl hydrazine (2,4 DNPH) and the standard vitamin [17]. The deproteinized extract or standard was incubated for 1.5 h with a solution of 2,4-DNPH (3%), CuSO_4_ (0.05%), thiourea (0.4%), and H_2_SO_4_ (9 N) at 37 °C. At the end of the incubation period, H_2_SO_4_ was added and the absorbance of the colored product was recorded at 520 nm after 30 min.

### HPLC analysis for identification and quantification of phenolics

Using specific phenolic standards, the BVJ phenolics were identified and quantified at 284 nm using Agilent 1260 Infinity HPLC series (Agilent Technologies, Palo Alto, CA, USA) [18]. The BVJ (20 μL) was separated by a ternary linear elution gradient on a 100 mm × 4.6 mm Kinetex EVO C18 column and eluted using 0.2% H_3_PO_4_, methanol, and acetonitrile.

### Evaluation of the antioxidant properties of BVJ

The antioxidant properties of BVJ, DMSA, and BVJ-DMSA (160:1) were evaluated in vitro using different methods, including total antioxidant capacity (TAC), ABTS^+^ radical cation decolorization, β-carotene-linoleate bleaching, and ferric reducing power. In all of these assays, except the TAC, different concentrations (0.25–0.01 mg/mL) of the samples were used.

The TAC has been determined at 695 nm using a mixture of 28 mM sodium phosphate, 4 mM ammonium molybdate, and 0.6 M H_2_SO_4_ [19]. The results were expressed as BHT equivalents in mg/g of juice using the BHT calibration curve. The ABTS^+^ radical cation-decolorization ability was assessed by reducing and decolorizing the blue-green chromophore ABTS^.+^ by the studied samples to ABTS [20]. The absorbance of the extract or control (no reduction) was read at 734 nm and the percentage of ABTS^+^ inhibition was calculated using the following equation:

$$ \mathrm{Radical}\ \mathrm{inhibition}\ \left(\%\right)=\left({\mathrm{A}}_{\mathrm{Control}}-{\mathrm{A}}_{\mathrm{Sample}}/{\mathrm{A}}_{\mathrm{Control}}\right)\times 100. $$

The β-carotene bleaching assay was determined to evaluate the anti-lipid peroxidation activity of the studied samples (BVJ, DMSA, or BVJ-DMSA) [21]. The ability of samples to prevent β-carotene bleaching in the emulsion of β-carotene, linoleic acid, and Tween-80 was detected at 490 nm. The absorbance (*a, b*) was recorded instantly and after 180 min (*t*), respectively then the degradation rate (DR) of the sample, control (water), and standard (BHT), was calculated using the equation: [DR = ln(*a*/*b*) × (1/*t*)]. Then the antioxidant ability was calculated as % of inhibition using the equation: antioxidant activity (%) = (DR_Control_ − DR_Sample_/ DR_Control_) × 100 to calculate the IC_50_ values (a concentration that inhibits the β-carotene bleaching by 50%). Ferric reducing power has been assessed using the potassium ferricyanide-ferric chloride method [22]. In this assay, each of the DMSA, single or combined extract or Asc was incubated with phosphate buffer (pH 6.6, 0.2 M) and potassium ferricyanide (1%) at 50 °C for 20 min. The mixture was acidified with 10% trichloroacetic acid (TCA) and mixed with 1% ferric chloride to produce a blue color solution with a maximum absorbance at 700 nm. Then the IC_50_ value (the concentration of the sample or Asc that gives 0.5 of absorbance) was calculated.

### Animals and experimental protocol

Seventy-two male albino rats weighing 70 ± 5 g obtained from Theodor Bilharz Research Institute, Giza, Egypt. The animals were housed in stainless cages under standard laboratory conditions of 12 h light/dark cycle, 55 ± 5% air humidity, and 30 °C and received a standard laboratory diet and tap drinking water for 2 weeks, as an adaptation period. All animal methodology was accomplished following the Institutional Animal Care and Use Committee (IACUC) and approved via the Committee of Animal Care and Use in Alexandria University (ethical approval reference number: AU-04200516301).

The animals have been randomly divided into 6 groups (twelve rats in each) as elucidated in (Fig. 2 I). The hepatotoxicity was induced in rats by ip injection of Pb (40 mg/kg b.w.) for 8 days,as elucidated before with modification [23]. Rats were orally administered with BVJ (1 g/kg b.w.) before, during, and after Pb injection, while DMSA (50 mg/kg b.w.) was orally intaken after Pb administration. The treatment protocol and the studied groups were designed as described in Fig. 2 I. After termination of the experimental period (31 days), rats were sacrificed under carbon dioxide euthanasia in compliance with the euthanasia recommendations in the Guide for the Care and Use of Laboratory Animals. Blood was obtained by cardiac puncture and one portion was collected in a heparinized tube for determination of the blood Pb level while the other portion was allowed to clot for 30 min to separate serum samples. Liver tissues were excised immediately, washed with cold saline, and small pieces were fixed in 10% formalin for histopathological examination. The remaining liver tissue was divided into two portions and kept at − 80 °C for the molecular and biochemical analyses.

### Blood analysis

The Pb concentration in the HNO_3_/H_2_O_2_ digested blood and liver samples has been quantified by atomic absorption spectrometry (Varian, model spectr AA 240, Mulgrave, Australia) [24]. In addition, the liver function parameters (albumin, ALP, ALT, and AST) and kidney function parameters (urea and Creatinine) were assessed in serum using the specific kits following the manual protocol.

### Determination of lipid peroxidation and antioxidant indices

The level of lipid peroxidation, GSH, total antioxidant efficiency, and activity of superoxide dismutase (SOD), glutathione peroxidase (GPX), catalase, and glutathione S-transferase (GST)α-1 were determined in the liver homogenate. The liver was homogenized in a phosphate buffer (pH 7) and a clear supernatant was obtained for the analyses after centrifugation at 6000 rpm for15 min. The lipid peroxidation level was determined by quantification of the TBA reactive substances (TBARS) level [25]. The GSH content was assessed by the reduction and cleavage of DTNB via GSH sulfhydryl group yielding a yellow-colored product with a maximum absorbance at 412 nm [26]. The total antioxidant efficiency of the liver tissue homogenates was assessed by the ABTS^+^ radical cation-decolorization method [20]. The method determines the degree of the disappearance of the blue-green color at 734 nm upon the reduction of the ABTS^+^ radical to ABTS by the homogenate antioxidants. For the preparation of the ABTS^+^ radical, 7 mM ABTS was incubated with 140 mM potassium persulphate at 25 °C for 16 h in the dark. The total antioxidant activity which expressed as μmol BHT equivalent/g liver was calculated using the BHT calibration curve.

The activity of Cu/Zn SOD was determined by quantifying the ability to inhibit the pyrogallol (20 mM) autoxidation at 420 nm [27]. One unit of activity is defined as the amount of the enzyme that inhibits the autooxidation reaction by 50%. The GPx activity was assessed colorimetrically using GSH and cummen hydrogen peroxide as substrates [28]. Catalase activity was determined spectrophotometrically by recording the cleavage of hydrogen peroxide at 240 nm [29]. The activity of GST was determined colorimetrically using p-nitrobenzyl chloride as a substrate [30]. In addition, the gene expression of GST α-1was determined by reverse transcription-quantitative polymerase chain reaction (RT-qPCR, BIO-RAD Laboratories, Inc., USA). Therefore, the total RNA was isolated from 100 mg of liver tissue by the RNA extraction kit, following the instruction protocol, then the purity and concentrations of the RNA samples were assessed by Nano-spectrometry (BioDrop μLITE, Cambridge, UK). The cDNA of the internal control (glyceraldehyde-3-phosphate dehydrogenase, GAPDH) and target genes were synthesized and amplified in a one-step reaction using specific forward and reverse primers as follow: GST α1 (forward); *5′-GCA TCA GCT TGC CCT TCA-3′*, GST α1 and (reverse); *5′-AAA CGC TGT CAC CGT CCT -3′*, GAPDH (forward); *5′-AGC CCA GAA CAT CAT CCC TG-3′*, GAPDH (reverse); *5′-CAC CAC CTT CTT GAT GTC ATC-3′*. The relative expression was measured by the RT-qPCR cycling program of one reverse transcription cycle (45 °C for 10 min) followed by polymerase activation (95 °C for 2 min). Then the denaturation (95 °C for 5 s), annealing (60 °C for 10 s), and extension (72 °C for 5 s). The expression of GST α-1 gene was calculated by the comparative Ct method (threshold cycle number at cross-point between amplification plot and threshold). The CT value was normalized to that of GAPDH according to the manufacturer’s protocol and the change in the gene expression was calculated using the 2^−ΔΔCT^ equation.

### Assessment of total protein content

The total protein level in liver homogenate and serum was determined using the Lowery method at 650 nm [31]. The values of the protein level in liver homogenate have been used to calculate the specific activity of the antioxidant enzymes as unit activity/mg of protein.

### Determination of the inflammatory biomarkers

The level of both interleukin-6 (IL-6) and nitric oxide (NO) were assessed in the liver tissue of all the studied groups. The protein level of IL-6 was determined using a specific ELISA kit following the instruction protocol. Griess reagent (0.1% naphthyl ethylenediamine dihydrochloride, 1% sulfanilamide, and 2% phosphoric acid) used to quantify the NO level colorimetricallyat 490 nm [32].

### Assessment of hepatic DNA fragmentation

The degree of DNA fragmentation was assessed spectrophotometrically as shown previously with some modifications [33]. In brief, 25 mg of liver tissue was homogenized in phosphate buffer “pH 7.0” then 250 μl of DNA lysis buffer “TTE” (1 M Tris-HCI pH 8, 0.2% Triton X-100, and 0.5 M EDTA) was added. Lysed cells were centrifuged for 10 min at 15000 rpm (4 °C). Then 0.5 mL of TTE solution was mixed with pellet and 50 μl of ice-cold NaCl (5 M) was vigorously mixed with supernatant prior to DNA precipitation with isopropanol. The DNA was washed via70% ethanol, dissolved in deionized water-RNase solution, and then incubated at 37 °C for 2 days. The absorbance (OD) was read at 260 nm using a nanodrop spectrophotometer then DNA fragmentation (%) was determined according to the equation: [(OD_supernatant_/OD_supernatant_ +  OD_pellet_) ] × 100

### Drug combination index (CI) analysis

The drug and food combination may offer greater (synergistic), lesser (antagonistic), or no new (additive) outcome compared to the single agent. This new result can be investigated by calculating the CI value (the predictable value divided by the observed value). The predictable value for BVJ-DMSA was calculated by the summation of the BVJ and DMSA half values for each studied in vitro antioxidant assay. While for other tested parameters, the predictable value is determined as [(observed value for BVJ)/(control value)] X [(observed value for DMSA)/ (control value)] X (control value) [34]. From the obtained value, the effect can be evaluated as synergism (CI < 1), antagonism (CI > 1), or additive (CI = 1) [35, 36].

### Histopathological study

The histopathology was done using the routine protocol. After fixation of the liver specimens, they have been embedded in paraffin wax and cut into thin slices with a thickness of 5 μm with a microtome then stained with hematoxylin and eosin for examination [37].

### Statistics

All data are expressed as mean ± standard error (SE). The statistical analysis was performed by SPSS (Statistical Package for Social Sciences) software version 25.0 usingone-way analysis of variance (ANOVA). The post hoc analysis using Dunnett’s test was followed and the values of *p* < 0.05 were considered as statistically significant. Heat map plots were obtained by ClustVis web server (https://biit.cs.ut.ee/clustvis/) [38].

## Results

### BVJ constituents

**Fig. 1:**
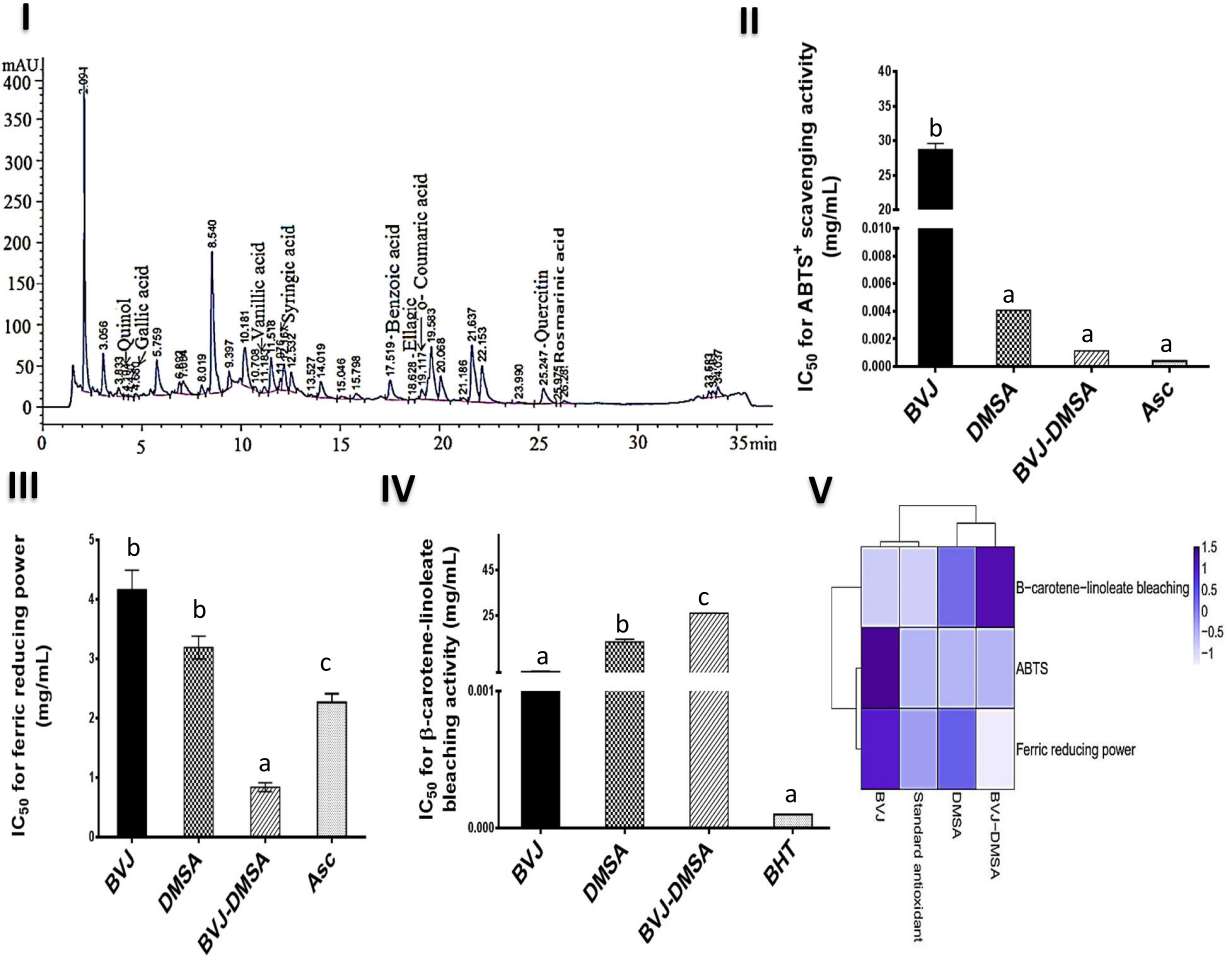
HPLC analysis and in vitro antioxidant activities of *Beta vulgaris* juice (BVJ), dimercaptosuccinic acid (DMSA), and their combination (BVJ-DMSA) compared to ascorbic acid (Asc) or butylated hydroxytoluene (BHT). (**I**) HPLC analysis of BVJ (**II**) ABTS^+^ radical scavenging activity (**III**) ferric reducing power (**IV**)β-carotene-linoleate bleaching activity (**V**) heat map plot of the in vitro antioxidant outcomes, the deep blue color refers to the higher IC_50_ value and the light color refers to the lower value. Results are shown as mean ± SE (*n* = 3). Different letters for the same parameter are significantly different at *p* < 0.05

Table 1 represents the constituents of BVJ, which included significant quantities of phenolics (flavonoids, Anthocyanins, Tannins), betalains and vitamin C. The HPLC analysis identified twelve phenolic compounds in BVJ (Fig. 1 I, Table 1) using known phenolic standards by comparing their particular retention times.

**Table 1:** Composition of some constituents in *Beta vulgaris* juice (BVJ)

Content	Constituents
1823.000 ± 87.000	Phenolics (μg 4-HCA Eq/ g BVJ)
14,187.000 ± 216.000	Flavonoids (μg RU Eq/ g BVJ)
88.394 ± 0.063	Anthocyanins (μg Cy-3-glc Eq./g BVJ)
96.526 ± 9.717	Tannins (μg catechin Eq/ g BVJ)
5730.000 ± 40.000	Total betalains (μg/g BVJ)
3680.000 ± 70.000	Betacyanins (μg/ g BVJ)
2040.000 ± 50.000	Betaxanthins (μg/ g BVJ)
105.666 ± 30.819	Vitamin C (μg/ g BVJ)
HPLC analysis of Phenolic compounds (μg g^− 1^ BVJ)	
16.021	Quinol
ND	Caffeine
881.732	Quercitin
ND	Rutin
35.074	Rosmarinic acid
132.339	Benzoic acid
ND	Ferulic acid
0.386	Ellagic acid
1031.673	Syringic acid
0.867	Gallic acid
35.297	Vanillic acid
121.048	o- Coumaric acid

Results are presented as Mean ± SE (*n* = 3). *HCA* Hydroxyl cinnamic acid, *RU* Rutin*, Cy-3-glc* Cyanidin-3-glucoside, *Eq* Equivalent, *ND* Not detected.

### In vitro antioxidant activities of BVJ and DMSA

Graphs II-IV in Fig. 1 revealed the in vitro antioxidant activities of BVJ, DMSA, and BVJ-DMSA compared to Asc or BHT. BVJ showed higher (*p* < 0.05) IC_50_ values for ABTS^+^ radical scavenging activity than the DMSA value (Fig. 1 II). Compared to Asc, BVJ showed significant (*p* < 0.05) lower power and the comparable impact was shown by DMSA. Concerning ferric reducing power (Fig. 1 III), both BVJ and DMSA had the same potency that was significantly (*p* < 0.05) smaller than Asc. The β-carotene-linoleate bleaching activity of BVJ was significantly (*p* < 0.05) more than DMSA and both exhibited significant (*p* < 0.05) lower activity than BHT (Fig. 1 IV). On the other hand, the combination of BVJ and DMSA (BVJ-DMSA) revealed synergistic (CI < 1) ABTS^+^ radical scavenging and ferric reducing power activities (Table 2) with similar or higher potency compared to Asc, respectively. However, the β-carotene-linoleate bleaching activity of BVJ-DMSA exhibited antagonistic (CI > 1) influence and lower potency than BHT. The heat map plot (Fig. 1 V) clustered these in vitro antioxidant results, where the deep blue color shows higher IC_50_ value (lower potency) and the light color indicates the lower value (higher potency).

**Table 2:** The combination index (CI)^*^ values for the *Beta vulgaris* juice (BVJ) and meso-2,3-dimercaptosuccinic acid (DMSA) mixture on the tested parameters

Parameters	CI	Effect
**In vitro** ***antioxidant models***		
ABTS (mg/mL)	0.000 ± 0.000	Synergistic
β-carotene bleaching (mg/mL)	3.764 ± 0.122	Antagonistic
Ferric reducing power (mg/mL)	0.245 ± 0.026	Synergistic
***Liver function parameters***		
ALT (U/L)	0.866 ± 0.072	Synergistic
AST (U/L)	0.545 ± 0.148	Synergistic
ALP (U/L)	0.626 ± 0.129	Synergistic
Albumin (mg/dl)	0.893 ± 0.107	Synergistic
Total protein (g/dl)	1.006 ± 0.062	Additive
***Kidney function markers***		
Urea (mg/dl)	0.798 ± 0.067	Synergistic
Creatinine (mg/dl)	0.725 ± 0.077	Synergistic
***Lead concentration***		
Blood lead level (μg/dl)	0.037 ± 0.012	Synergistic
Hepatic lead Level (μg/g tissue)	0.062 ± 0.013	Synergistic
***Oxidative stress markers***		
Lipid peroxidation (pmol/g tissue)	0.641 ± 0.050	Synergistic
GSH (mg/g tissue)	0.864 ± 0.019	Synergistic
TAC (μg BHT Eq/g tissue)	0.969 ± 0.136	Synergistic
SOD (IU/mg protein)	0.624 ± 0.144	Synergistic
GPX (IU/mg protein)	0.655 ± 0.099	Synergistic
Catalase (IU/mg protein)	0.7663 ± 0.162	Synergistic
GST (IU/mg protein)	1.1262 ± 0.226	Antagonistic
GST α1 (expression fold)	1.814 ± 0.600	Antagonistic
***Inflammatory parameters***		
NO (mmol/g tissue)	0.677 ± 0.119	Synergistic
IL-6 (pg/g tissue)	0.402 ± 0.100	Synergistic
DNA fragmentation (%)	0.844 ± 0.084	Synergistic

^*^CI value of < 1 shows synergistic effect; > 1 shows antagonistic effect; = 1 shows additive effect. *TAC* Total antioxidant capacity, *HCA* Hydroxycinnamic acid, *ABTS* 2,2- azino-bis(3-ethylbenzthiazoline-6-sulfonic acid, *BHT* Butylated hydroxytoluene, *ALT* Alanine aminotransferase*, AST* Aspartate aminotransferase, *ALP* Alkaline phosphatase, *GSH* Reduced Glutathione, *SOD* Superoxide dismutase, *GPX* Glutathione peroxidase, *GST* Glutathione S-transferase, *NO* Nitric oxide, *IL-6* Interleukin-6

### Liver and kidney biomarkers levels before and after administration of BVJ, DMSA, and their combination in Pb-intoxicated rats

**Fig. 2:**
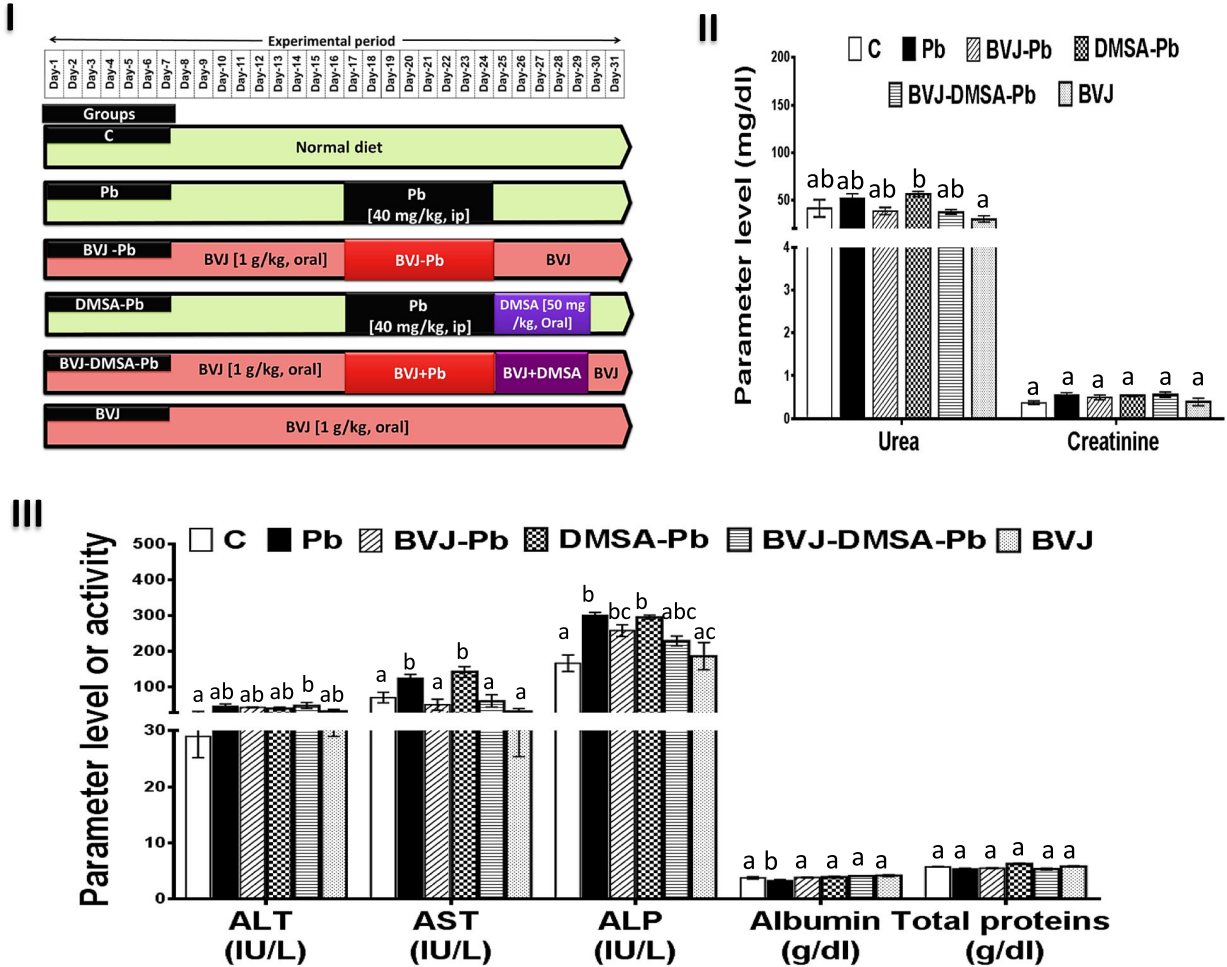
The improving effects of *Beta vulgaris*juice (BVJ), dimercaptosuccinic acid (DMSA), and their combination (BVJ-DMSA) on the serum kidney and liver biomarkersof Pb-intoxicated rats. (**I**) the experimental design (**II**) urea and creatinine levels (**III**) activities and levels of liver function parameters. Results are shown as mean ± SE (*n* = 8). Different letters for the same parameter are significantly different at *p* < 0.05.***C***, control; ***ip***, intraperitoneal; ***ALT***,alanine aminotransferase; ***AST***, aspartate aminotransferase; ***ALP***, alkaline phosphatase

As shown in (Fig. 2 II, III), Pb administration did not significantly change the serum levels of kidney biomarkers (urea and creatinine), as well as ALT activity and total protein level by about 25.43, 47.17, 59.77, and 6.80%, respectively, compared to the control samples. Nevertheless, the activity of AST and ALP elevated significantly (*p* < 0.05) by approximately 75.76 and 80.09%, respectively. In contrast, the level of albumin reduced significantly (*p* < 0.05) by about 12.76%.

The treatment with BVJ, before, during, and after Pb administration changed the level of urea (25.86%), creatinine (10.26%), and total protein (2.66%) and the activity of ALT (6.22%) non significantly compared to Pb group. However, BVJ significantly (*p* < 0.05) prevented Pb-induced elevation in AST activity (59.12%) and depletion in albumin level (16.82%), but it cannot avoid the elevation in ALP activity (14.29%). On the other hand, the treatment with DMSA showed a non-significant elevation in the level of urea (8.47%), creatinine (1.65%), and total proteins (16.79%) and the activity of ALT (13.24%), AST (13.66%), and ALP (1.79%) relative to rats in Pb group. However, it significantly raised and normalized the level of albumin by 19.07%.

The combination of BVJ and DMSA (BVJ-DMSA) showed a non-significant change in the level of urea (27.79%), creatinine (2.01%), and total proteins (0.48%) and the activity of ALT (4.68%) and ALP (23.95%) as compared to Pb group. However, the level of albumin was significantly (*p* < 0.05) raised by 25.49% and the activity of AST was significantly decreased by 51.08% compared to the Pb group. The synergy testing (Table 2) revealed a synergistic (CI < 1) effect of BVJ-DMSA for all the kidney and liver biomarkers except for total proteins it showed additive effect (CI = 1).

The administration of BVJ alone to rats for 31 days demonstrated a non-significant change in the level of urea (27.54%), creatinine (5.12%), ALT (15.52%), AST (54.34%), ALP (11.51%), albumin (10.77%), and total proteins (1.08%) as compared to control group.

### Depleting impact of BVJ, DMSA, and their combination to blood and hepatic Pb levels in Pb-intoxicated rats

**Fig. 3:**
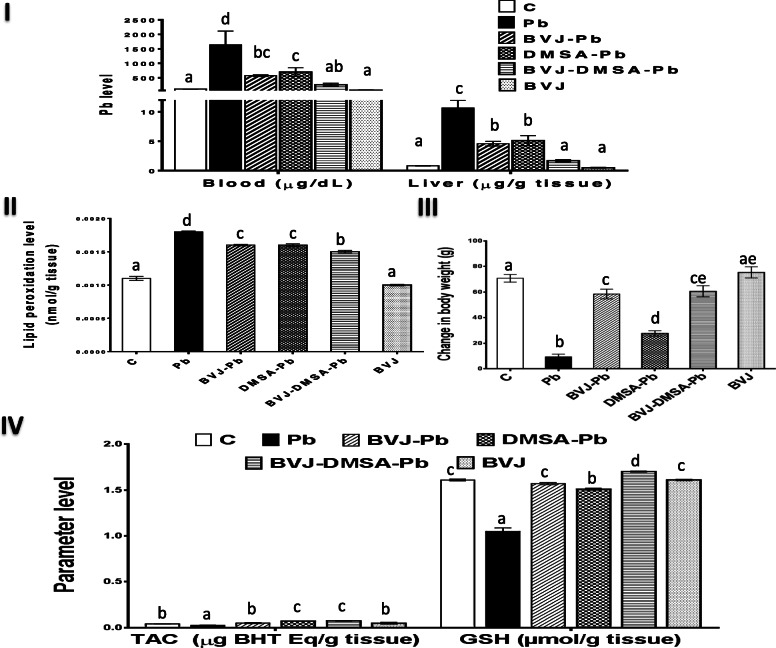
The alleviating influences of *Beta vulgaris*juice (BVJ), dimercaptosuccinic acid (DMSA), and their combination (BVJ-DMSA) on level of Pb in blood and liver and Pb-induced oxidative stress in the liver tissue of Pb-intoxicated rats. (**I**) the blood and liver Pb levels (**II**) lipid peroxidation level (**III**) change in body weight (**IV**) total antioxidant capacity (TAC) and reduced glutathione (GSH) level. Results are shown as mean ± SE (*n* = 8). Different letters for the same parameter are significantly different at *p* < 0.05.***BHT***,Butylated hydroxytoluene;***C***, control

As shown in Fig. 3 I, the blood and hepatic Pb levels were significantly (*p* < 0.05) elevated after Pb administration by 1413.86 and 1187.27%, respectively, compared to the control group. Administration of BVJ, before, during, and after Pb administration significantly prevented the elevation in blood and hepatic Pb levels by 64.97 and 56.97%, respectively. Similarly, the treatment with DMSA significantly decreased both Pb levels by 57.85 and 51.98%, respectively. The combination of BVJ and DMSA (BVJ-DMSA-Pb group) synergistically (CI < 1) reduced the Pb level in blood and liver (Table 2) by 84.09% compared to Pb group. On the other hand, the administration of BVJ alone slightly reduced blood and hepatic Pb levels by 33.18 and 40.61%, respectively, relative to the control group.

### The hepatoprotective role of BVJ, DMSA, and their combination on Pb-induced OS

**Fig. 4:**
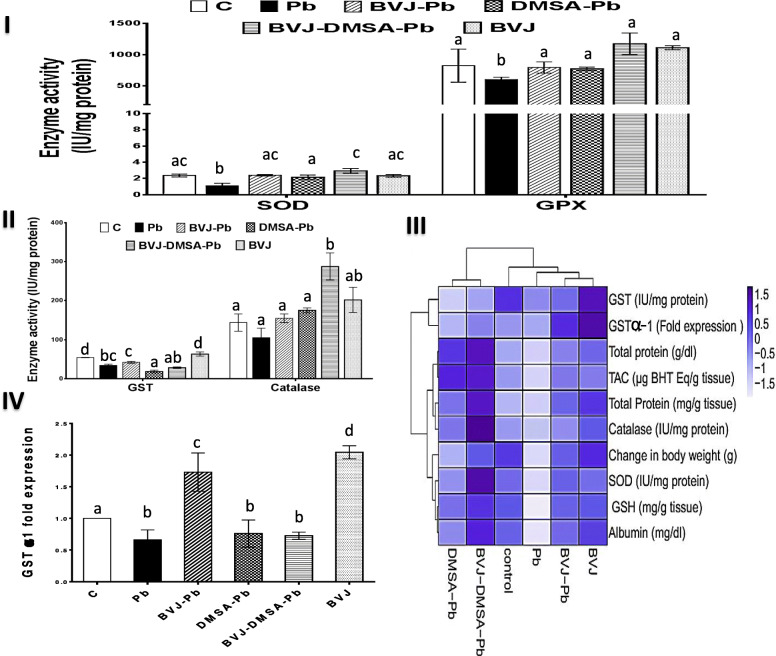
The ameliorating effects of *Beta vulgaris*juice (BVJ), dimercaptosuccinic acid (DMSA), and their combination (BVJ-DMSA) on the activity of hepatic antioxidant enzymesin Pb-intoxicated rats. (**I**) the activities of hepatic superoxide dismutase (SOD) and glutathione peroxidase (GPX) (**II**) the activities of hepatic glutathione-S transferase (GST) and catalase (**III**) Heat map diagram clustering all the testedbiochemical parameters that were depleted by Pb. The deep blue color indicates the higher values, activities or fold expression and the light color refers to the lower ones (**IV**) the fold expression of GST α1.Results are shown as mean ± SE (*n* = 8). Different letters for the same parameter are significantly different at *p* < 0.05.***C***, control; ***TAC***, total antioxidant capacity; ***GSH***, reduced glutathione

The graphs (II-IV) in Fig. 3 and graphs (I-III) in Fig. 4 elucidated the findings of Pb-induced OS markers and changes in the body weight of rats in the studied groups. The outcomes were clustered as a heat map plot (Fig. 4IV). Administration of Pb induced significant (*p* < 0.05) increase in the level of lipid peroxidation (Fig. 3 I) by 63.63% relative to control rats. Also, it induced a significant decline in the body weight (Fig. 3 III), TAC and GSH levels (Fig. 3 IV) by 86.73, 39.02, and 34.78% respectively. Moreover, the activity of SOD, GPX (Fig. 4 I), GST, and catalase (Fig. 4 II), as well as the gene expression of GST α1 (Fig. 4 IV), were depleted significantly (*p* < 0.05) after Pb administration by 53.81, 27.02, 37.61, 26.96, and 33.70%, respectively.

The intake of BVJ, before, during, and after Pb administration significantly (*p* < 0.05) prevented the elevation in the lipid peroxidation level and the decrease in the experimental animal’s body weight by 11.11 and 522.02%, respectively, compared to Pb group. In addition, the administration of BVJ has significantly avoided the depletion in the level of TAC and GSH and the activity of SOD, GPX, and GST by 96%, 49.52, 120.18%, 32.54, and 23.53%, respectively. However, it prevented the suppression of catalase activity non-significantly, and significantly (*p* < 0.05) up-regulated the gene expression of GST α1 by 47.32 and 160.93%, respectively, compared to the Pb group.

Likewise, DMSA administration significantly reduced the degree of lipid peroxidation and increased the body weight of the animals tested by 11.11 and 194.79%, respectively, relative to the Pb group. Also, DMSA intake significantly (*p* < 0.05) raised the level of both TAC (43.81%) and GSH (43.81%) and the activity of SOD (96.33%) and GPX (28.94%). However, it non-significantly elevated the activity of catalase (66.67%) and the gene expression of GST α1 (14.63%) and significantly reduced the activity of GST (46.09%) compared to the Pb group.

The combination of BVJ and DMSA showed a synergistic (CI < 1) effect for all the studied OS parameters, except for the GST activity and GST α1 expression, which showed antagonistic effects (Table 2). The administration of this combined form significantly reduced the lipid peroxidation level and elevated the animal’s body weight by 16.67 and 544.68%, respectively as compared to Pb group. In addition, this administration significantly (*p* < 0.05) increased the level of TAC (192%) and GSH (62.09%),the activity of SOD (168.81%), GPX (95.24%), and catalase (173.81%). However, it non-significantly affected the activity of GST (16.68%) and the gene expression of GST α1 (9.35%) relative to the Pb group.

Administration of BVJ alone non-significantly changed the level of lipid peroxidation (9.09%), rat body weight (6.45%), TAC (17.07%), and GSH (0%). Similarly, it non-significantly changed the activity of SOD (1.27%), GPX (34.94%), GST (16.20%), and catalase (40.21%). However, it significantly (*p* < 0.05) upregulated the gene expression of GST α1 by 104.30% relative to the control group.

### The hepatoprotective role of BVJ, DMSA, and their combination on Pb-induced inflammation and DNA fragmentation

**Fig. 5:**
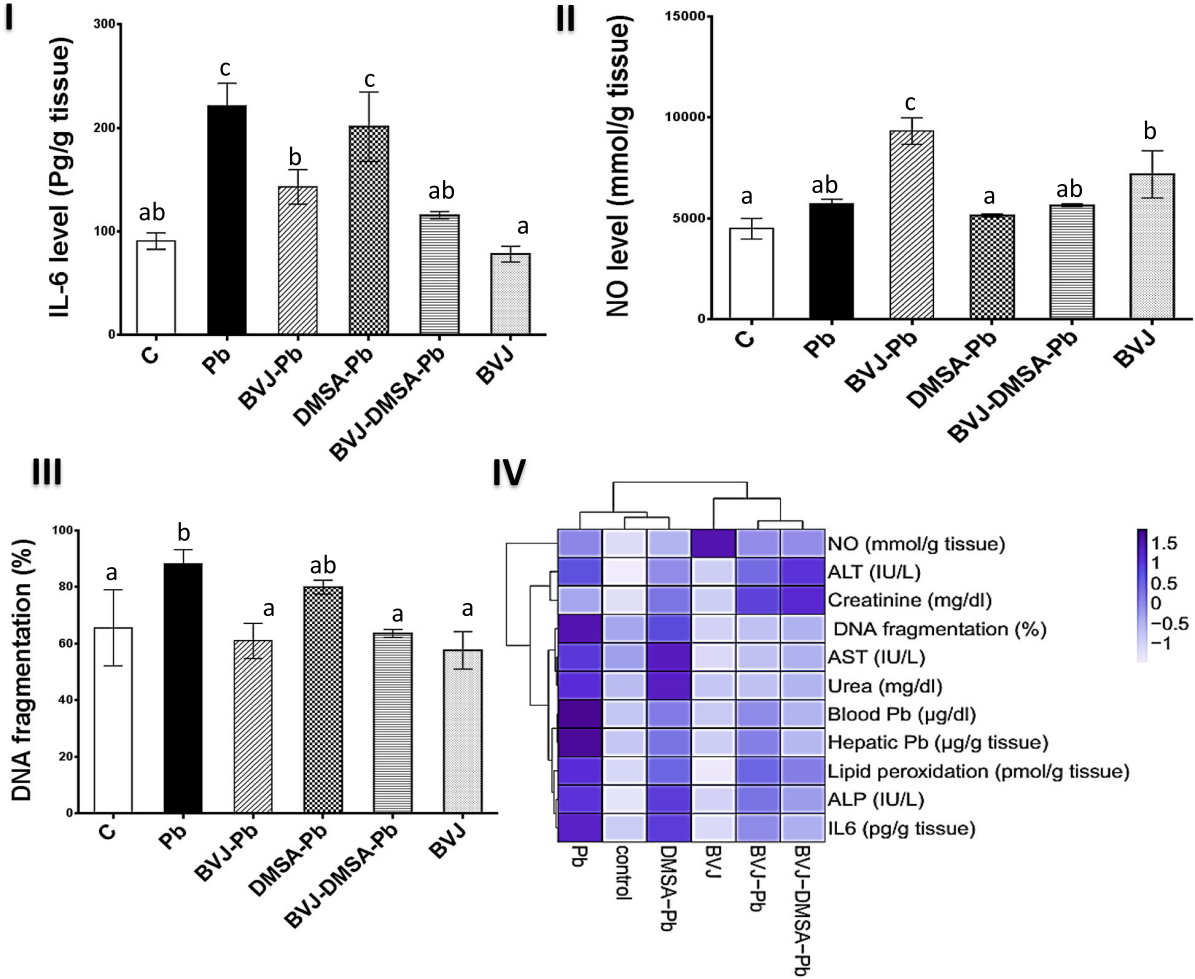
The ameliorating effects of *Beta vulgaris* juice (BVJ), dimercaptosuccinic acid (DMSA), and their combination (BVJ-DMSA) on Pb-induced inflammation and DNA fragmentation in liver tissue of Pb-intoxicated rats. (**I**) interleukin (IL)-6 levels (**II**) nitric oxide (NO) levels (**II**I) the % of DNA fragmentation level (**IV**) Heat map diagram clustering all the tested biochemical parameters that were elevated by Pb. The deep blue color indicates the higher values or activities and the light color refers to the lower ones. Results are shown as mean values (*n* = 8). Different letters for the same parameter are significantly different at *p* < 0.05.*C*, control; *ALT*,alanine aminotransferase; *AST*, aspartate aminotransferase; *ALP*, alkaline phosphatase

Fig. 5(I-III) revealed the effect of BVJ, DMSA, and their combination on the inflammatory markers (IL-6 and NO) and DNA fragmentation. These parameters and others that were elevated by Pb were hierarchically clustered by the heat map diagram (Fig. 5IV). The results showed that the administration of Pb caused a significant elevation in the level of IL-6 (143.74%) and DNA fragmentation in hepatocytes (34.61%), but non-significantly affect the NO level (27.16%).

Administration of BVJ, before, during, and after Pb administration extremely depleted the IL-6 level (35.29%) and DNA fragmentation (30.88%). In contrast, it significantly (*p* < 0.05) elevated the NO level by 63.11%relative to Pb group. While DMSA treatment non-significantly depleted any of these parameters by about 9%. On the other hand, the combination between BVJ and DMSA revealed a synergistic (CI < 1) anti-inflammatory effect and a synergistic suppressing effect on DNA fragmentation (Table 2). This combination significantly (*p* < 0.05) reduced the hepatic level of IL-6 and DNA fragmentation by 27.90% and slightly (1.21%) changed the NO level in liver tissue. However, the administration of BVJ alone for 31 days altered the hepatic level of IL-6 and DNA fragmentation non-significantly by 13.61 and 12.09%, respectively, and substantially (59.67%) raised the NO level relative to control rats.

### Histopathological changes of the liver in the Pb-intoxicated rats before and after administration of BVJ, DMSA, or their combination

**Fig. 6:**
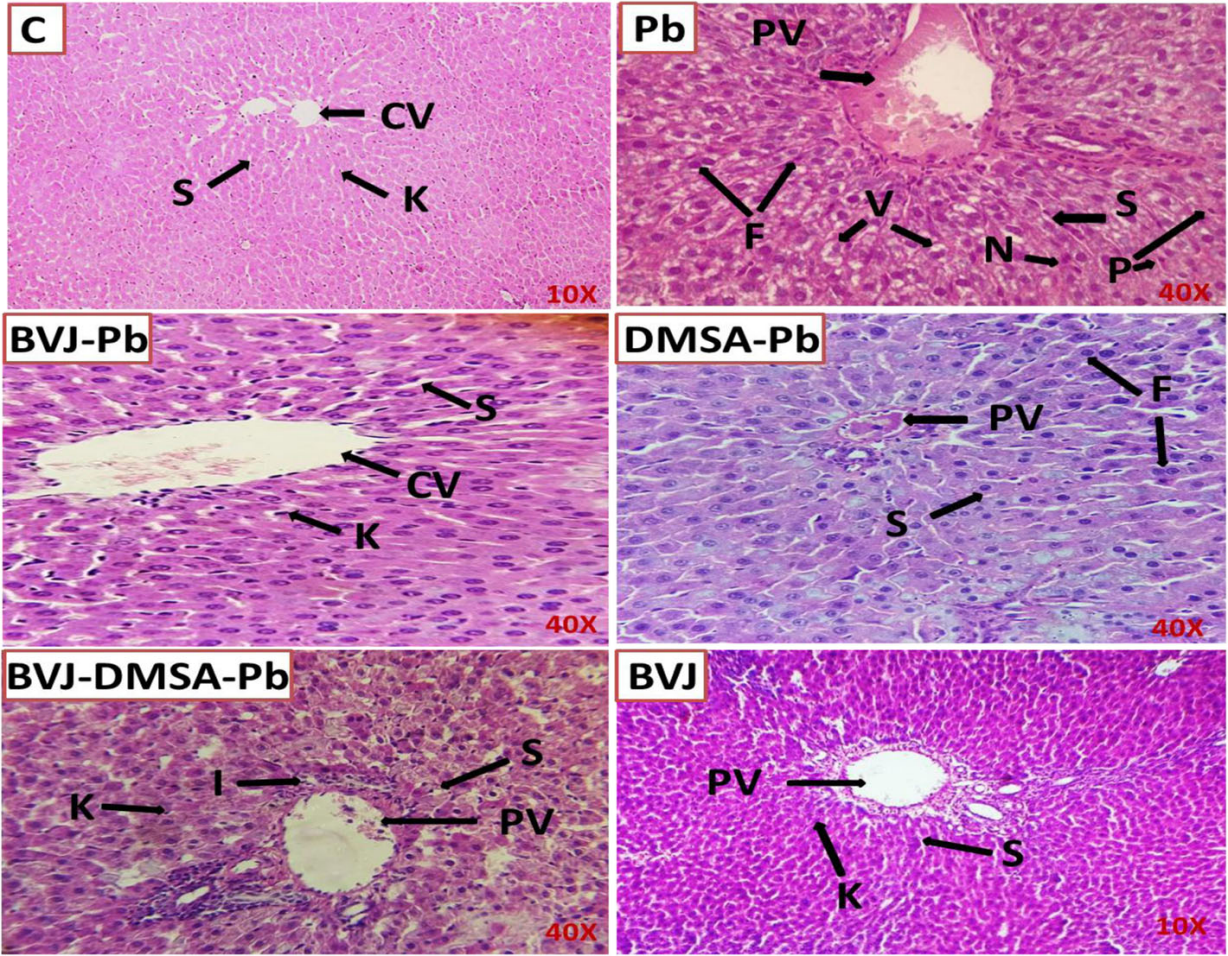
Histopathological changes in the liver of rats in all the studied groups. *BVJ*, *Beta vulgaris* juice; *C*, control;*CV*,central vein; *DMSA*, dimercaptosuccinic acid;*F*,feathery degeneration of hepatocytes; *I*,focal lytic inflammation; *K*, kupffer cells; *N*, single cells necrosis; *P*, pyknotic nuclei; *PV*, portal vein; *S*, sinusoids; *V*, vaculation; 10*x*, *40x*, magnification of the image

The control liver tissues displayed typical morphological appearance with normal polygonal (pentagonal and hexagonal) hepatocytes that are separated by asymmetrical sinusoids containing Kupffer cells (Fig. 6). This normal architecture was disrupted after Pb administration. Hence, the normal cordlike arrangement of the hepatocytes was damaged and cells revealed vacuolated cytoplasm, pyknotic nuclei, and single-cell necrosis. In addition, there was an infiltration of inflammatory cells and congestion of the portal vein and sinusoids. The administration of BVJ, before, during, and after Pb, protected from these damage effects caused by Pb and there was only congestion of the central vein with normal hepatocytes and sinusoids. However, the treatment with DMSA demonstrated feathery degenerative hepatocytes with congested central vein and sinusoids. The combination of BVJ and DMSAshowed normal hepatic tissue architecture with only focal lytic inflammation. On the other hand, the administration of BVJ alone for 31 days had no adverse effect on the hepatic architecture.

## Discussion

The fruits, vegetables, and herbs are rich in bioactive compounds with various health benefits such as antioxidative, atherosclerosis, anticarcinogenic, and antimutagenic inhibitory potentials [11, 39, 40]. The current study revealed the presence of multiple bioactive constituents in BVJ, including phenolics (flavonoids, anthocyanins, and tannins), betalains, and vitamin C (Table 1). These compounds have redox power and can scavenge and neutralize free radicals. Therefore, BVJ has ferric reducing power, scavenge ABTS^+^, and prevent β-carotene bleaching (Fig. 1). These results were following the previous studies that confirm the antioxidant activities of BV [41]. Moreover, DMSA revealed antioxidant potency against the studied free radicals (Fig. 1) that probably owed to its structure that contains dithiol compound (−SH groups) [42]. DMSA revealed higher potency towards ABTS radical while BVJ showed higher β-carotene-linoleate bleaching ability and both had the same ferric reducing power. The combination of DMSA and BVJ had synergistic efficacy (CI < 1) relative to those individuals, except for the β-carotene-linoleate bleaching study (Table 2). This will enhance the quality and therapeutic potential of this combination.

The present study demonstrated the hepatoprotective role of BVJ, DMSA, and their combination on the Pb-induced hepatotoxicity in rats. The liver is known to be the largest storehouse of Pb [1] as well as, one of the main organs concerned with storing, biotransformation, and removal of Pb. This may result in an elevation in the activity of AST and ALP and a decrease in serum albumin levels, which may be indicative of hepatic damage [43]. Pb caused OS in the liver by upregulating lipid peroxidation causing loss of membrane integrity and elevation of the ROS. This will influence the redox state of the cell and antioxidant indices. GSH is one of the cellular antioxidants used to detoxify peroxides and various electrophilic compounds using GST and GPX. In addition, GSH neutralized the radicals-generated from Pb metabolism and make chelation and detoxification of it [3]. By accumulating Pb and increasing the Pb level over time, this will cause exhaustion of the antioxidant enzymes including SOD, catalase, and GPX. In addition, these enzymes have been inactivated by Pb due to inhibition of their functional –SH groups. Also, Pb can chelate selenium, which is an important cofactor for GPX activity [3]. The GST activity decreased after Pb administration that may be owed to increase the production of peroxides after Pb metabolism that leads to exhaustion of the enzyme. Also, the gene expression of GSTα1 was downregulated after Pb intake. This may be related to the ROS production that caused oxidative damage for the DNA in the liver [44]. All of these damage effects induced by Pb caused depletion of the TAC in liver tissue. Moreover, Pb administration caused hepatic inflammation by raising the level of early mediators (IL-6) and secondary mediators (ROS). IL-6 is a pro-inflammatory cytokine that induces neutrophil influx, prostaglandin synthesis, and T and B lymphocytes activation [45]. In addition, ROS production was considered to be an essential factor in inducing inflammation due to the crosstalk between ROS and NF-кB, one of the most essential inflammatory pathways. Induction of OS and inflammation in the cell upon Pb intake is toxic and can trigger both apoptotic and necrotic cell death [46]. Therefore, the DNA fragmentation level was massively elevated following Pb injection (Fig. 5 III). All of these damage effects are clearly observed in the morphology of the liver tissue of the Pb-intoxicated rats (Fig. 6).

The present research found that BVJ administration before, during and after Pb intoxication significantly lowered blood and hepatic Pb levels and clearly enhanced the blood profile of these rats. Moreover, this extract capable of returning the homeostasis and the balance between the hepatic free radicals content and the antioxidant status to ameliorate the Pb induced oxidative stress. This can be indicated by lowering the lipid peroxidation level and increasing the antioxidant indices, including TAC, GSH, SOD, GPX, and GST α1. These parameters have not only been enhanced, but most of them have also been normalized (Figs. 3, 4). However, the BVJ did not have any impact on the activity of both hepatic catalase and GST and significantly increased the gene expression of GST α1. The improvement in body weight change in the rats of this group reflects this enhanced impact. The ability of BV extracts to alleviate the OS induced by different toxicants was studied before [39]. The potent antioxidant role of BVJ may be related to its ingredients, including flavonoids, phenolic acids, and betalains (Table 3). Hence, gallic, vanillic, syringic, ellagic, rosmarinic, benzoic, and o-coumaric acids proved their potency as antioxidants. Also, flavonoids [47], as well as betalains [41], can improve the OS state and return the cellular homeostasis. However, the up-regulation of GST α1 expression may be linked to the existence of betalains that can induce the hepatic phase II enzymes, like GST α1, as studied before [48]. Additionally, administration of BVJ prevented the elevation of the inflammatory mediators like IL-6, but upregulated the NO level in the liver tissues (Fig. 5). The anti-inflammatory activity of BVJ constituents [47] may be the cause of its depleting effect on IL-6 level (Table 3). While the elevation of NO level may be owed to its nitrate content as reported before [49]. The elevation of NO with the depletion of IL-6 and OS can confirm the absence of the pathological action of NO. Hence, the previous studies suggested that the elevation of NO after BV administration is associated with increasing the production of muscle power through an unclear mechanism [49]. Therefore, BVJ administration, before, during, and after Pb can protect from both the oxidative and inflammatory stress in the hepatic tissue. This may be the cause for the normalization of the DNA fragmentation level in the hepatic tissue of rats administered this extract. Hence, several studies proved the implication of OS and inflammation in cell death [50]. The present study found also that all of the studied biochemical parameters were in harmony with the histopathological outcomes (Fig. 6).

**Table 3:** Summary of the hepatoprotective effect of *Beta vulgaris* juice (BVJ) phytochemicals against Pb toxicity

***Phytochemical compound***	***The hepatoprotective mechanism***	***Reference***
Betalains	- antioxidant and free radicals scavenging. up-regulation of GST α1 expression	[41, 48]
Rutin	- scavenge and neutralize free radicals.	[41]
Flavonoids	- scavenge and neutralize free radicals. Significant prevention of IL-6 production. Improvement of antioxidant indices and decrease lipid peroxidation level.	[47]
Gallic acid		
Syringic acid		
Vanillic acid		
Ellagic acid		
Rosmarinic acid		
Benzoic acid		
o- Coumaric acid		

Additionally, the current study proved the safety of BVJ intake for 31 days. Hence, there were no pathological changes in the studied OS and inflammatory indices in hepatic tissues as well as the studied blood profile. Only a substantial increase in NO level and significant elevation of GST α1 gene expression were observed in the rats of this group. This may be owed to its nitrate richness and its betalains content, respectively as discussed earlier. The hepatic histology of BVJ-treated rats alone disclosed ordinary architecture which confirmed the safety of this extract. These findings will increase the protective efficiency and the importance of BVJ.

On the other hand, this study reported the therapeutic effect of DMSA on Pb-induced hepatic damage. The effectiveness of DMSA appears almost as BVJ potency in most of the studied markers. It was able to ameliorate the Pb-induced OS and inflammation. This may be related to its structure containing the reduced sulfhydryl groups that enable it to act as a potent antioxidant molecule and improved the hepatic OS and in turn the inflammatory response and DNA fragmentation [42]. Although the level of IL-6 did not show any improvement, which implies the presence of the hepatic inflammation. This has been seen in the histology of the liver tissue of these rats, in addition to feathery hepatocytes, which may be owed to the few side effect of DMSA as studied before [8]. Such findings were consistent with the previous research by Ercal et al. [51].

The present study also studied the anti-toxicity effect of the combination between both BVJ and DMSA (BVJ-DMSA-Pb). This combination revealed a synergistic antioxidant and anti-inflammatory effects in most of the studied parameters as indicated by the CI value (Table 2). Several combination studies of DMSA and other antioxidants were previously investigated and gave remarkable results against Pb-induced hepatotoxicity, such as the results of Flora et al. [52]. Here, in addition to the synergistic efficiency of this combination, it also reduced the observed side effect of DMSA on the hepatic tissue. Hence, the histopathological results of this combination showed no pathological changes in the hepatic structure (Fig. 6). The obtained synergism between DMSA and BVJ may be related to their antioxidant properties. As several studies have shown synergistic antioxidant and anti-inflammatory impacts after the combination of different antioxidant extracts or drug and extract [18, 34, 53]. However, the exact mechanism for this synergism needs further investigations and analyses.

## Conclusion

In summary, our results clearly revealed that BVJ has potent efficiency in the protection from Pb-induced hepatotoxicity via reducing the accumulation of Pb in blood and liver and by preventing Pb-induced oxidative and inflammatory stress. Likewise, the administration of DMSA (the known Pb chelator) showed enhancing effects from Pb toxicity, but produced certain adverse impacts on the liver. However, the intake of BVJ and DMSA mixture after Pb injection showed synergistic antioxidant and anti-inflammatory influences with minimal side effects on the hepatic tissue. Therefore, BVJ is a successful extract in protection from Pb-induced hepatotoxicity and its combination with DMSA exerted a potent therapeutic effect against this toxicity.

## Data Availability

The data that supported this article are available in Tables 1, 2 and, Figs. 1, 2, 3, 4, 5, 6. The data sets analyzed during the present study are available from the corresponding author on a reasonable request.

## Abbreviations

*ABTS* 2,2: Azino-bis(3-ethylbenzthiazoline-6-sulfonic acid
*ALP*: Alkaline phosphatase
*BV*: *Beta vulgaris*
*BHT*: Butylated hydroxytoluene
*BVJ*: BV juice juice
*CI*: Combination index
Cy-3-glc: Cyanidin-3-glucoside
*DMSA*: Meso-2,3-dimercaptosuccinic acid
*DR*: Degradation rate
*GSH*: Glutathione
GST: Glutathione-s transferase
GPX: Glutathione peroxidase
4-HCA: 4-hydroxycinnamic acid
*IL-6*: Interleukin-6
*NO*: Nitric oxide
*OS*: Oxidative stress
*RNS*: Reactive nitrogen species
*ROS*: Reactive oxygen species
*SOD*: Superoxide dismutase
*TBA*: Thiobarbituric acid
*TBARS*: Thiobarbituric acid reactive substances
*TAC*: Total antioxidant capacity
